# Significance of poor performance status after resection of colorectal liver metastases

**DOI:** 10.1186/s12957-017-1306-1

**Published:** 2018-01-05

**Authors:** Peter Strandberg Holka, Sam Eriksson, Jakob Eberhard, Magnus Bergenfeldt, Gert Lindell, Christian Sturesson

**Affiliations:** 1 Department of Clinical Sciences Lund, Surgery, Skane University Hospital, Lund University, Lund, Sweden; 2 Department of Clinical Sciences Lund, Oncology, Skane University Hospital, Lund University, Lund, Sweden; 3 Department of Surgery, Skåne University Hospital, S-221 85 Lund, Sweden

**Keywords:** Colorectal neoplasms, Hepatectomy

## Abstract

**Background:**

Performance status (PS) is known as one of the strongest prognostic factors for survival in metastatic colorectal cancer patients. The aim of the present study was to analyze factors associated with poor PS assessed after resection for colorectal liver metastases and the impact on survival.

**Methods:**

All patients undergoing curative resection for colorectal liver metastases between 2010 and 2015 in a single center were reviewed retrospectively.

**Results:**

A total of 284 patients were included, out of whom 74 patients (26%) presented with a postoperative PS WHO > 2 precluding administration of adjuvant chemotherapy. These patients had a shorter recurrence-free survival (*P* = 0.002) and shorter overall survival (*P* < 0.001). Multivariable analysis showed that patients with PS > 2 after surgery had higher preoperative ASA score, had a higher frequency of major complications after surgery, and had more frequently synchronous liver and lung metastases. PS was found to be the strongest independent factor predicting survival (hazard ratio 0.45). When patients with postoperative PS > 2 developed recurrent disease (54 of 74), 43 (80%) received tumor specific treatment.

**Conclusions:**

Patients with postoperative PS > 2 who did not receive adjuvant chemotherapy had decreased recurrence-free and overall survival after liver resection for colorectal liver metastases. After recurrence, a large majority of these patients had had improvement in PS allowing for administration of tumor specific treatment.

## Background

Colorectal cancer (CRC) liver metastatic disease is a significant clinical problem. Up to 25% of patients with colorectal cancer are diagnosed with synchronous liver metastases and a similar number develop metachronous metastases [1]. When possible, surgical resection is the treatment of choice [1, 2]. The 5-year survival rates after resection range from 15 to 74% [3, 4], the wide distribution mainly due to differences in patient selection, a development over time with improved chemotherapeutic and biological agents, improved surgical techniques and a change in multimodality approach [5].

However, about two thirds of the patients develop disease recurrence within 2 years after resection [6]. Adjuvant chemotherapy aims to treat micro-metastatic disease to reduce the risk of relapse [7]. The strategy of perioperative chemotherapy has proved to increase the time of progression-free survival [8]. In addition, adjuvant therapy after surgery alone confers a survival benefit [9–12]. Recently, the failure of patients to receive adjuvant chemotherapy due to poor performance status (PS) and complications after liver resection has been shown to have a negative impact on survival [13, 14]. PS is a scoring system quantifying the impact of disease on a patient’s well-being and is known as one of the strongest prognostic factors for survival in metastatic CRC patients [15, 16]. The definitions of PS are shown in Table 1 [17]. The aim of the present study was to analyze factors associated with poor PS after resection for colorectal liver metastases and the impact on survival. In case of disease recurrence, the aim was to evaluate the PS of patients and to which extent patients received tumor specific treatment.

**Table 1:** Definitions of performance status

Grade	WHO performance status
0	Fully active, able to carry on all pre-disease performance without restriction
1	Restricted in physically strenuous activity but ambulatory and able to carry out work of a light or sedentary nature, e.g., light house work, office work
2	Ambulatory and capable of all self-care but unable to carry out any work activities; up and about more than 50% of waking hours
3	Capable of only limited self-care; confined to bed or chair more than 50% of waking hours
4	Completely disabled; cannot carry on any self-care; totally confined to bed or chair
5	Dead

## Methods

Medical records of all patients undergoing resection of colorectal liver metastases between 2010 and 2015 at a single center were reviewed. All patient data were retrieved retrospectively. Liver resection was performed as previously described [18]. A major resection was defined as resection of ≥ 3 Couinaud’s segments, and 30-day morbidity was classified according to Clavien-Dindo [19]. Postoperative poor PS was defined as a PS WHO > 2. Adjuvant chemotherapy was defined as chemotherapy administered within 90 days after liver surgery. Indication for adjuvant therapy was considered when a R0 or R1 resection was performed and patients presented with a postoperative PS WHO 0–2. Synchronous disease was defined as when liver metastases were diagnosed during the radiological staging before resection of the primary. The overall and recurrence-free survival were recorded.

The study protocol was approved by the regional ethics committee.

### Statistical analysis

Results were expressed as median and interquartile range. Mann-Whitney *U* test was used to compare continuous data and *χ*^2^ test for categorical data. The Kaplan-Meier method was used to estimate the recurrence-free survival and overall survival. The log-rank test was used to compare the importance of postoperative PS. To analyze the effect of risk factors for adverse survival outcome, Cox regression analysis was used and hazard ratios with 95% confidence intervals were calculated. Factors with a *P* < 0.1 on univariable Cox regression analysis were included in the multivariable analysis. *P* < 0.05 was considered statistically significant. Statistical analysis was performed using IBM SPSS Statistics version 22 (IBM, Armonk, NY, USA).

## Results

A total of 284 patients were resected for colorectal liver metastases and form the study cohort. All patients had a postoperative consultation with an oncologist within 5–7 weeks after resection for PS evaluation and planning of adjuvant chemotherapy. All patients had PS WHO 0–2 before liver resection. Seventy-four patients (26%) presented with PS WHO > 2 after surgery. These patients did not receive any adjuvant chemotherapy. The remaining 210 patients (74%) received adjuvant chemotherapy, either as adjuvant treatment alone or as a complement to preoperative chemotherapy. Adjuvant chemotherapy was oxaliplatin-based (*n* = 152), 5-fluorouracil alone (*n* = 36) or irinotecan-based (*n* = 15). Seven patients received a combination of two or more cytostatic regimens. Patients with adjuvant chemotherapy received a median of 7 (5–8) postoperative chemotherapy cycles.

The patient characteristics for the groups with postoperative PS WHO 0–2 and WHO > 2 are shown in Table 2. No patient had tumor progression as reason for PS WHO > 2. For patients with PS WHO 0–2 receiving adjuvant chemotherapy, treatment was initiated 50 (42–64) days after resection.

**Table 2:** Patient characteristics for patients with postoperative WHO performance status 0–2 vs. WHO performance status > 2

	WHO performance status, 0–2 *N* = 210	WHO performance status, > 2 *N* = 74	*P* value
Male gender	125 (59.5%)	51 (68.9%)	0.152
Age, years	68 (62–73)	68 (62–73)	0.224
Smoking	49 (23.3%)	10 (13.5%)	0.073
Diabetes mellitus	22 (10.5%)	9 (12.2%)	0.689
Body mass index (kg/m^2^)	25 (23–28)	25 (23–27)	0.969
ASA grade 3–4	53 (25.2%)	31 (41.9%)	0.007
Preoperative albumin (g/l)	38 (35–40)	38 (35–40)	0.452
Preoperative creatinine (μmol/l)	73 (63–83)	80 (66–95)	0.052
Rectal primary	71 (35.5%)	25 (36.8%)	0.851
Primary T4	45 (24.7%)	12 (19.7%)	0.420
Node positive primary	116 (63.7%)	47 (77.1%)	0.056
Synchronous disease	116 (58.0%)	30 (44.1%)	0.047
Synchronous lung and liver metastases	11 (5.7%)	11 (16.4%)	0.006
> 1 tumour	125 (60%)	49 (66%)	0.353
Tumour size > 50 mm	22 (10.5%)	8 (10.8%)	0.936
Preoperative chemotherapy	123 (58.6%)	41 (55.4%)	0.635
Preoperative chemotherapy cycles	5 (4–6)	4 (3–5)	0.600
Preoperative chemotherapy > 6 cycles	16 (7.7%)	8 (10.8%)	0.416
Operation time (hours)	5 (3–6)	5 (3–7)	0.251
Operative bleeding (ml)	400 (200–600)	400 (200–900)	0.145
Major resection	94 (44.8%)	33 (44.6%)	0.980
Hospital stay (days)	7 (6–8)	7 (6–10)	0.214
Morbidity (Clavien-Dindo ≥ 3)	19 (9.0%)	16 (21.6%)	0.005

Data are presented as number (percentage) or median (interquartile range)

*ASA* American Society of Anesthesiologists

The median follow-up after liver resection was 33 months. During follow-up, 116 of 210 (55%) patients developed recurrence of their disease in the group with postoperative PS WHO 0–2. Ten patients (9%) did not receive any tumor-specific treatment after relapse due to poor PS. Fifty-four of 74 (73%) patients in the group with postoperative PS WHO > 2 suffered from recurrence. In this group, 11 of 54 patients (20%) did not receive tumor-specific treatment at recurrence due to poor PS (*P* = 0.032 as compared with the group WHO 0–2).

**Fig. 1:**
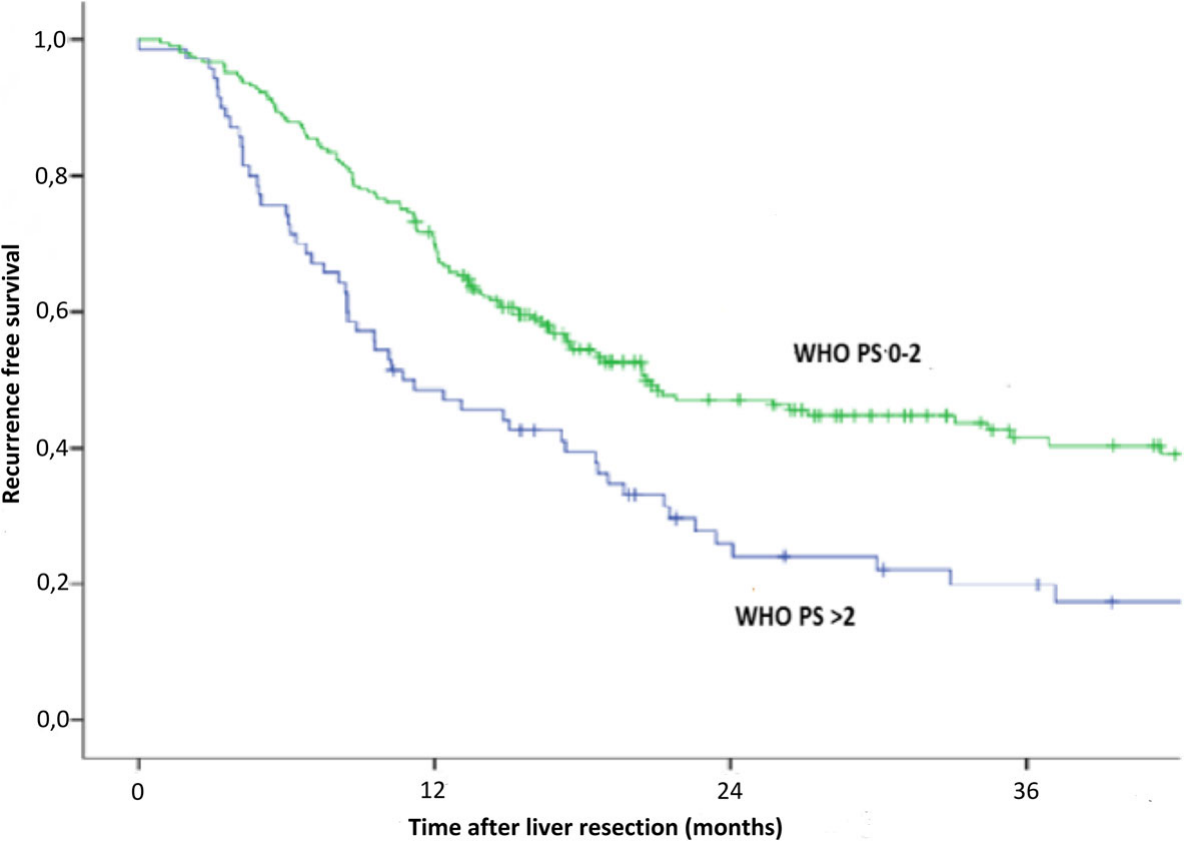
Kaplan-Meier estimate of recurrence-free survival (*P* = 0.002, log rank test). PS, performance status

**Fig. 2:**
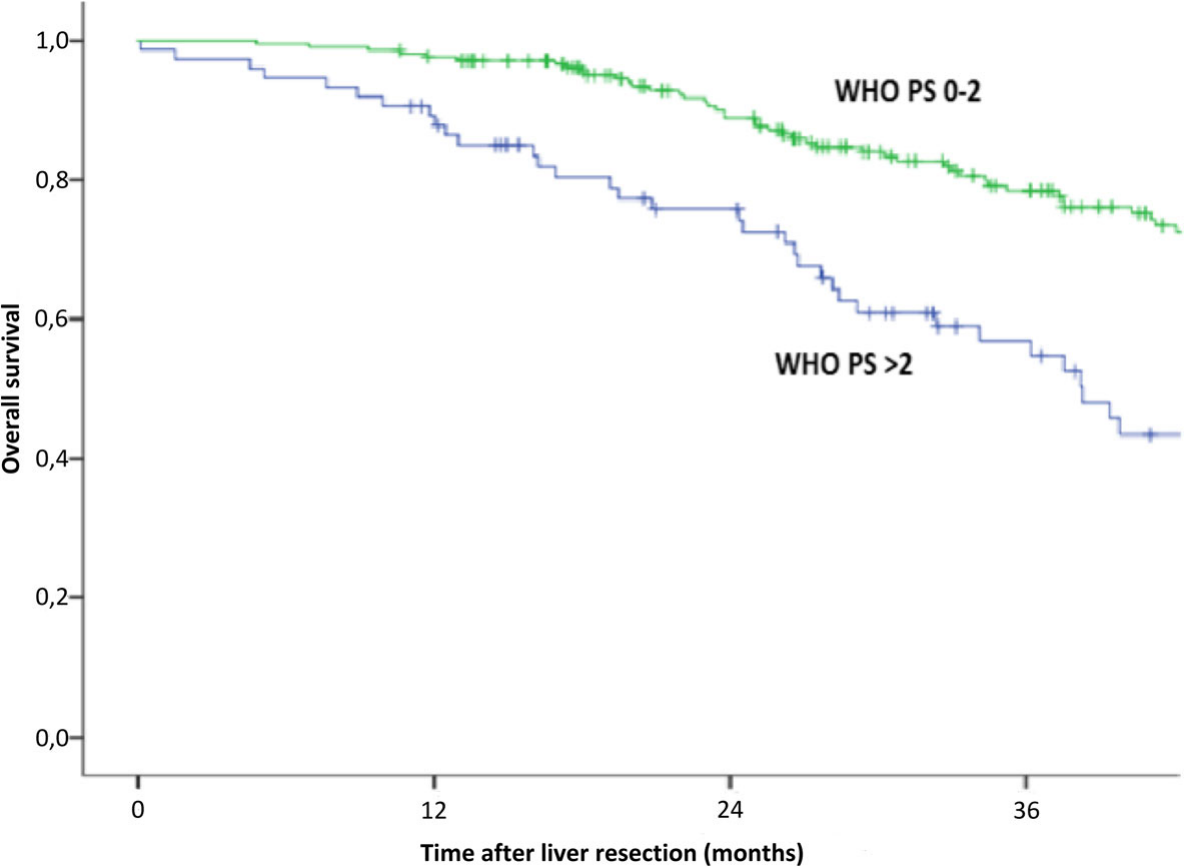
Kaplan-Meier estimate of overall survival (*P* < 0.001, log rank test). PS, performance status

Kaplan-Meier estimates of recurrence-free and overall survival are shown in Figs. 1 and 2. The results of the Cox proportional hazard analysis of the adverse risk factors for survival outcome are shown in Table 3.

**Table 3:** Cox proportional hazard analysis of risk factors for adverse survival outcome

	Univariate		Multivariate	
Variable	HR (95% CI)	*P* value	HR (95% CI)	*P* value
Male gender	1.38 (0.91–2.10)	0.125		
Age	1.00 (0.98–1.0)	0.816		
Smoking	1.02 (0.63–1.67)	0.931		
Diabetes mellitus	0.99 (0.53–1.86)	0.986		
Body mass index	0.99 (0.94–1.05)	0.841		
ASA grade 3–4	1.40 (0.93–2.11)	0.111		
Rectal primary	1.24 (0.83–1.87)	0.292		
Primary T4	1.63 (0.99–2.67)	0.053	1.40 (0.84–2.34)	0.197
Node positive primary	1.70 (1.02–2.84)	0.041	1.42 (0.83–2.46)	0.203
Synchronous disease	1.62 (1.08–2.45)	0.021	1.77 (1.05–2.96)	0.031
Preoperative chemotherapy	1.72 (1.15–2.59)	0.009	1.25 (0.75–2.08)	0.394
Preoperative chemotherapy > 6 cycles	1.57 (0.86–2.88)	0.142		
Major resection	1.16 (0.78–1.71)	0.461		
Tumour size > 50 mm	0.93 (0.48–1.79)	0.826		
Morbidity (Clavien-Dindo ≥ 3)	1.68 (1.0–2.83)	0.051	1.38 (0.70–2.70)	0.352
WHO performance status, 0–2 vs > 2	0.45 (0.30–0.68)	< 0.001	0.52 (0.32–0.86)	0.010

*HR* hazard ratio, *ASA* American Society of Anesthesiologists

## Discussion

In the present study, 26% of patients resected for colorectal liver metastases displayed a poor postoperative PS preventing administration of adjuvant chemotherapy. These patients had shorter overall and recurrence-free survival. In line with other studies [15], poor PS was the strongest independent factor for survival.

Patients with PS WHO > 2 had higher ASA score as a measure of preoperative medical comorbidity and a more advanced disease with synchronous liver and lung metastases. This group of patients was also to a greater extent affected by major postoperative complications (Clavien-Dindo ≥ 3). The reasons for change in PS are thus a combination of preoperative patient characteristics and postoperative adverse event. Because of poor PS, these patients did not receive oncological adjuvant chemotherapy. This finding is in line with previous studies reporting a failure to receive intended adjuvant chemotherapy of 13–37% of patients [8, 13, 14]. Postoperative morbidity has previously been shown to decrease the ability to tolerate adjuvant chemotherapy [13]. To enhance postoperative recovery, a fast-track protocol is used at our department since 2012, resulting in decreased length of stay but preliminary without impact on morbidity [18]. However, decreased morbidity after introduction of enhanced recovery protocols has been demonstrated [20]. In addition, laparoscopic liver resections are getting more widespread with the potential of lowering morbidity further [13, 21]. The impact on these measures on postoperative PS and the ability to tolerate adjuvant chemotherapy is still to be investigated.

Adjuvant chemotherapy was initiated a median of 7 weeks after liver resection. After resection of colon cancer primary, evidence exists that adjuvant chemotherapy should be administrated within 8 weeks after surgery for best survival benefit [22]. There is however no data supporting that there is a similar window of opportunity for starting adjuvant chemotherapy after liver resection. For a patient, in too poor general health to tolerate adjuvant chemotherapy on a postoperative consultation, the choice is between reassessing the patient after some time period and inhibiting adjuvant chemotherapy. No reassessment was done in the present study, much likely reflecting a general notion of an existing upper time limit for starting adjuvant chemotherapy extrapolated from treatment of primary colon cancer.

A significant number of patients in both WHO 0–1 and WHO > 2 groups developed recurrent disease. A vast majority of patients in the WHO 0–2 group with recurrence was given tumor-specific treatment. In the WHO > 2 group, 54 of 74 (73%) patients developed recurrent disease. Also, in this group, most patients (80%) received tumor specific treatment. These results also indicate that some of the patients who initially presented with poor PS after surgery, and therefore were not considered for adjuvant chemotherapy, could possibly improve their PS by reducing postoperative complications. In addition, a later postoperative assessment of PS may potentially increase the number of patients who could tolerate postoperative adjuvant treatment. Further studies are necessary to investigate the optimal time window for adjuvant chemotherapy after surgery for colorectal liver metastases.

Although extensively used, the effective role of adjuvant chemotherapy is still controversial.

The present study showed that patients presenting with poor postoperative PS not receiving chemotherapy after liver resection had a shorter recurrence-free survival and a shorter overall survival. Previously reported in two randomized studies, adjuvant chemotherapy was associated with a trend to better survival [9, 10]. The pooled data from both studies [11] support the assumption that adjuvant chemotherapy is associated with a longer progression-free survival as well as longer overall survival. Another large study [12] comparing resection of colorectal liver metastases with and without adjuvant chemotherapy showed that adjuvant chemotherapy prolongs postoperative survival. Liver resection and perioperative chemotherapy have been shown to result in a significant increase in disease-free survival when compared to surgery alone [8], although no difference in overall survival could be demonstrated [23].

Decreased recurrence-free survival and overall survival in patients with poor postoperative PS not receiving adjuvant chemotherapy in the present study could be due to patient selection. These patients had more medical comorbidity as well as more advanced disease in terms of synchronous lung metastases. In addition, no analysis of the influence of R0 or R1 status was made.

It is well known that PS is a strong prognostic factor for survival in patients with metastatic colorectal cancer [16]. There are also indications that patients with a good PS are the ones to benefit the most from adjuvant chemotherapy after resection of colorectal metastases [24]. Postoperative complications after resection of colorectal liver metastases have been associated with poor overall and recurrence-free survival, as well as delayed initiation of chemotherapy [14]. It seems therefore crucial to reduce postoperative complications, which possibly could be achieved by introducing enhanced recovery programs and shifting into more mini-invasive surgical techniques.

## Conclusions

The present study showed that a significant number of patients undergoing liver resection for colorectal liver metastases did not recover in time for the intended adjuvant chemotherapy. These patients had shorter overall and recurrence-free survival. However, a great majority of patients that presented with poor PS after surgery received palliative chemotherapy or other surgical treatment after disease recurrence.

## Abbreviations

ASA: American Society of Anesthesiologists
CRC: Colorectal cancer
PS: Performance status
